# Case report: Immunotherapy-based combination therapy achieving complete remission and prolonged survival in nasopharyngeal carcinoma with extensive bone marrow metastasis

**DOI:** 10.3389/fimmu.2024.1410250

**Published:** 2024-06-24

**Authors:** Xingran Wang, Ying Yuan, Yan Zhang, Guanglei Qiao, Shihong Chen, Wentao Huang, Jianjun Zhang

**Affiliations:** ^1^ Department of Oncology, Tongren Hospital, Shanghai Jiao Tong University School of Medicine, Shanghai, China; ^2^ Department of Clinical Laboratory, Tongren Hospital, Shanghai Jiao Tong University School of Medicine, Shanghai, China; ^3^ Department of Pathology, Tongren Hospital, Shanghai Jiao Tong University School of Medicine, Shanghai, China

**Keywords:** nasopharyngeal carcinoma, recurrence and metastasis, bone marrow, immunotherapy, chemotherapy

## Abstract

Nasopharyngeal carcinoma with bone marrow metastasis presents a rare and challenging clinical scenario associated with exceedingly poor prognosis. While standard treatment regimens offer limited efficacy and tolerability in such cases, individualized approaches are increasingly necessary. We present the case of a 64-year-old male diagnosed with recurrent nonkeratinizing undifferentiated nasopharyngeal carcinoma with extensive bone marrow metastasis (rTxN0M1). Treatment was initiated with immunotherapy-based combination therapy, consisting of pembrolizumab and low-dose cisplatin, which resulted in an initial response. Subsequently, there was a transition to standard-dose nab-paclitaxel-cisplatin chemotherapy in combination with pembrolizumab, followed by maintenance therapy with pembrolizumab plus fruquintinib. The patient achieved a sustained response with renormalization of tumor markers, imaging findings, and bone biopsies, resulting in complete remission. This case highlights the successful management of nasopharyngeal carcinoma with extensive bone marrow metastasis through an individualized treatment approach incorporating immunotherapy.

## 1 Introduction

Nasopharyngeal carcinoma (NPC) is a rare malignancy in most regions worldwide but exhibits a high incidence in southern China (1). Early-stage and locally advanced NPC are typically managed with radical radiotherapy, sometimes complemented by concurrent or induction chemotherapy (2). For NPC with distant metastasis, systemic therapy is the cornerstone of management. While there is no curative treatment available for patients with distant metastasis, the overall prognosis has improved due to the advancement of treatment regimens, transitioning from cisplatin-based combination chemotherapy to chemotherapy combined with immune checkpoint inhibitors (3–6).

To detect metastases in NPC, fluorine-18 fluorodeoxyglucose (FDG) positron emission tomography/computed tomography (FDG-PET/CT) is widely used due to its high diagnostic accuracy. A previous meta-analysis has indicated an overall accuracy exceeding 90% for the detection of nodal and distant metastases (7).

However, bone marrow metastasis from NPC is rare, and the prognosis for these patients is exceedingly poor (8–10). Due to the severe complications associated with bone marrow metastasis, patients are typically ineligible for standard first-line regimens. Individualized therapy is a sensible choice for such patients.

Here, we present a case of recurrent NPC with extensive bone marrow metastasis. The patient underwent immunotherapy-based combination therapy, resulting in a sustained response and prolonged survival.

## 2 Case presentation

**Figure 1:**
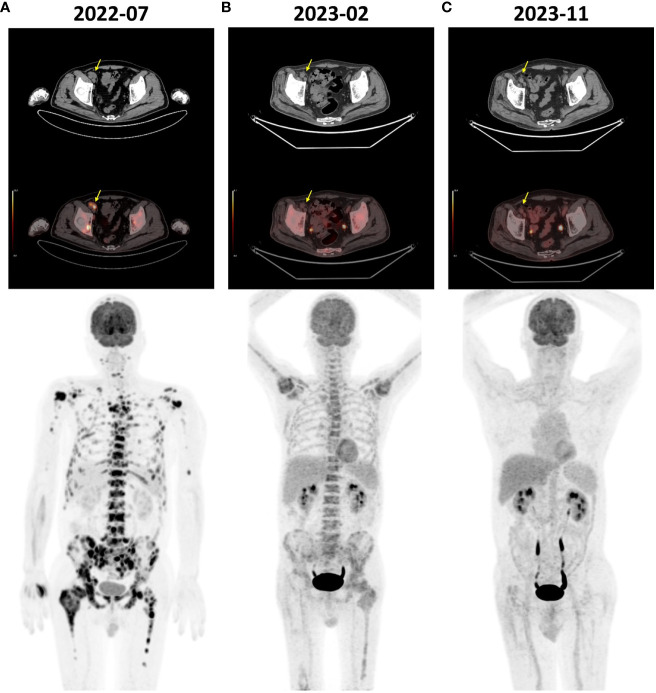
FDG-PET/CT images of the patient. **(A)** The pre-treatment scan (July 2022) revealed diffuse bone FDG uptake (SUVmax 15.6) and multiple lymph node enlargements adjacent to the right iliac vessels (yellow arrow) showing increased FDG uptake (SUVmax 13.8). **(B)** The scan before the 5th cycle of treatment (February 2023) revealed a significant reduction in bone FDG uptake (SUVmax 5.3) and regression of the enlarged lymph nodes (yellow arrow). **(C)** The scan conducted at the 14th month post-treatment initiation (November 2023) showed normal systemic bone FDG uptake and no evidence of disease. FDG, fluorine-18 fluorodeoxyglucose; PET/CT, positron emission tomography/computed tomography; SUVmax, maximum standardized uptake value.

A 64-year-old male patient presented with progressive lumbar and right hip pain and was admitted to our hospital on September 20, 2022. He was diagnosed with stage III nonkeratinizing undifferentiated NPC (cT3N2M0) 21 months prior. The *in situ* hybridization (ISH) test for Epstein-Barr virus-encoded RNA (EBER) showed a positive result. He received two cycles of cisplatin plus fluorouracil induction chemotherapy, followed by radical radiotherapy with concurrent cisplatin chemotherapy. Post-treatment evaluation confirmed complete remission. By July 2022, the patient experienced impaired mobility due to right hip pain. FDG-PET/CT revealed diffuse bone FDG uptake [maximum standardized uptake value (SUVmax) 15.6] with a pathological fracture of right femoral neck and multiple parailiac lymphadenopathies showing increased FDG uptake (SUVmax 13.8) (**Figure 1A**). On August 12, 2022, he underwent right hip tumor resection and artificial femoral head replacement in another hospital. Histological examination of the resected hip joint suggested undifferentiated squamous cell carcinoma invasion (EBER ISH positive). The level of plasma Epstein-Barr virus DNA (EBV-DNA) was below the limit of detection. Oncogenetic testing indicated a tumor mutational burden of 1.03 mutations per Mb unit, and a microsatellite stable status. Two weeks post-surgery, the patient developed anemia and thrombocytopenia, which worsened during subsequent follow-up. Consequently, the patient sought treatment at our center 39 days after the artificial femoral head replacement surgery.

**Figure 2:**
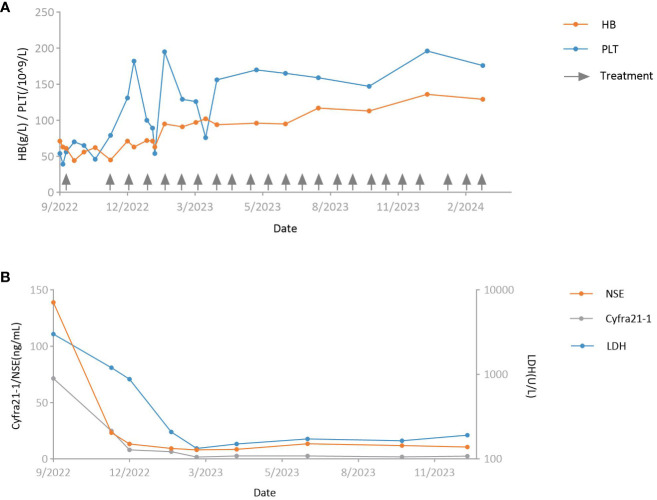
Curves of key laboratory tests. **(A)** Hemoglobin level (orange) and platelet count (blue). Arrows indicate the timing of antitumor treatment. **(B)** Serum levels of neuron-specific enolase (orange), cytokeratin 19 fragment (gray), and lactate dehydrogenase (blue). HB, hemoglobin; PLT, platelet; NSE, neuron-specific enolase; Cyfra21–1, cytokeratin 19 fragment; LDH, lactate dehydrogenase.

On admission, the physical examination revealed the patient was totally confined to bed due to limited mobility following surgery and weakness, with a low-grade fever and signs of anemia. Blood tests revealed a hemoglobin level of 71 g/L, a platelet count of 54*10^9/L, and a white blood cell count of 8.62*10^9/L (**Figure 2A**). Serum levels of cytokeratin 19 fragment (71.50 ng/ml) and neuron-specific enolase (139.00 ng/ml) were elevated, while the levels of other tumor markers were within the normal range (**Figure 2B**). Particularly noteworthy was the serum lactate dehydrogenase level, which was elevated to 3000 U/L (**Figure 2B**). The patient was initially treated with analgesics, nutritional support, empirical anti-infective therapy, subcutaneous denosumab for bone metastasis, and subcutaneous recombinant human thrombopoietin plus interleukin-11 for thrombocytopenia.

**Figure 3:**
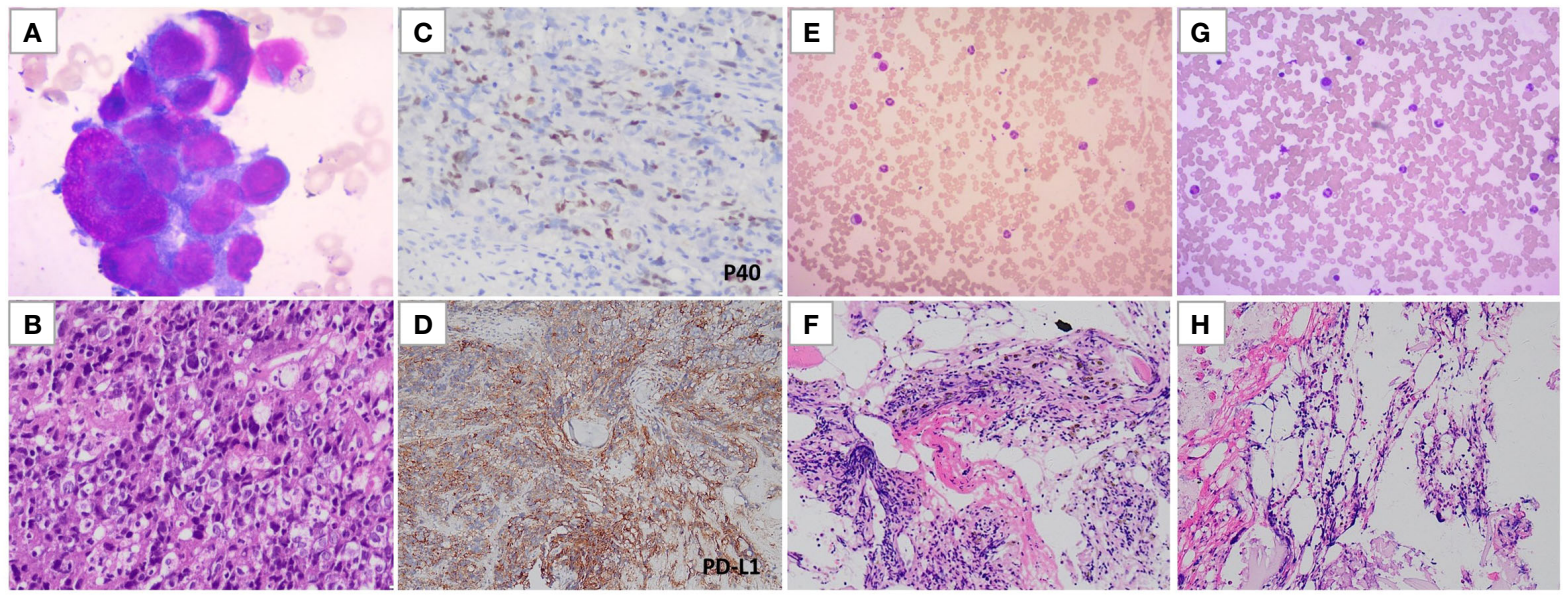
Hematoxylin-eosin (H&E) staining and immunohistochemical evaluation of the bone marrow aspiration and biopsy. **(A)** H&E staining of the pre-treatment bone marrow smear. **(B)** H&E staining of the pre-treatment bone marrow biopsy. **(C, D)** Immunohistochemical evaluation of P40 and PD-L1 for the pre-treatment bone marrow biopsy. **(E, F)** H&E staining of the bone marrow smear and biopsy obtained at the 12^th^ month post-treatment. **(G, H)** H&E staining of the bone marrow smear and biopsy obtained at the 14^th^ month post-treatment.

Considering the possibility of bone marrow metastasis, the patient underwent bone marrow aspiration and biopsy from the left posterior superior iliac spine on the third day of admission. The bone marrow smear revealed infiltration of malignant tumor cells (**Figure 3A**). The bone marrow tissue biopsy confirmed metastasis of undifferentiated carcinoma with elevated PD-L1 expression (combined positive score 80) (**Figures 3B-D**). The EBER ISH test of the bone marrow tissue biopsy was negative, but immunohistochemistry staining showed positive results for p40 and p63. The patient was diagnosed with bone marrow metastasis from NPC, necessitating urgent anti-tumor therapy. However, a repeat blood test revealed contraindications for chemotherapy, with a hemoglobin level of 70 g/L and a platelet count of 38*10^9/L (**Figure 2A**). Consequently, a tailored regimen was initiated, consisting of pembrolizumab (200 mg intravenous infusion on day 1) and low-dose cisplatin (40 mg intravenous infusion on day 2).

Over the next two months after starting treatment, the patient experienced recurrent episodes of declining hemoglobin and platelet count, with the hemoglobin dropping to a minimum of 44 g/L and the platelet count reaching a minimum of 46*10^9/L (**Figure 2A**). He received transfusions of red blood cell suspension and continued treatment for thrombocytopenia with recombinant human thrombopoietin. Fortunately, the patient experienced substantial pain relief, and tumor markers decreased significantly from pre-treatment levels (cytokeratin 19 fragment and neuron-specific enolase levels dropped to 24.90 ng/ml and 23.20 ng/ml, respectively) (**Figure 2B**). The patient received a second dose of pembrolizumab 60 days after the first cycle. Due to anemia and thrombocytopenia, cisplatin infusion was discontinued in the second cycle. After cycle 2, the patient’s platelet count and hemoglobin level gradually recovered after a transient decline (**Figure 2A**). On day 18 of the second cycle, the hemoglobin level increased to 71 g/L, and the platelet count rose to 131*10^9/L. The cytokeratin 19 fragment level dropped to 8.05 ng/ml, and neuron-specific enolase decreased to within the normal range. Serum lactate dehydrogenase level also decreased significantly to 878 U/L (**Figure 2B**).

Subsequently, the regimen was escalated to standard dosing, which consisted of pembrolizumab (200 mg intravenous infusion on day 1 every 3 weeks), nab-paclitaxel (125mg/m^2^ intravenous infusion on days 1 and 8 every 3 weeks), and cisplatin (75mg/m^2^ intravenous infusion on days 1 every 3 weeks). Before the start of cycle 5, a tumor evaluation was conducted. Compared to the pre-treatment level, FDG-PET/CT revealed a significant reduction in bone FDG uptake and regression of the lymph nodes adjacent to the right iliac vessels, indicating a partial response (**Figure 1B**). Treatment was continued with the same regimen. After six cycles of pembrolizumab plus standard-dose chemotherapy, chemotherapy was discontinued, and pembrolizumab was continued as maintenance therapy. However, considering the bone FDG uptake identified in the latest FDG-PET/CT scan, low-dose fruquintinib (3 mg on days 1–14 every 3 weeks) was added to prevent disease progression. The maintenance therapy has been continuously administered until present without any severe adverse events.

At months 12 and 14 post-treatment initiation, bone marrow aspiration biopsies were performed at the left and right posterior superior iliac spine, respectively, revealing no tumor cells on pathology (**Figures 3E-H**). Furthermore, at the 14th month, FDG-PET/CT scan showed a normal systemic bone FDG uptake and normal-sized lymph nodes adjacent to the right iliac vessel, confirming complete remission (**Figure 1C**). During the course of the disease, the results of plasma EBV-DNA tests were all below the limit of detection. To date, the patient has achieved a progression-free survival (PFS) of 18 months.

## 3 Discussion

NPC is endemic to southern China (1). Distant metastasis occurs in 6% to 8% of initially diagnosed patients and 15% to 30% of those who have received treatment, posing a significant challenge to oncologists (2, 11). Before the era of immunotherapy, cisplatin-based chemotherapy served as the standard first-line treatment for these patients. The GEM20110714 phase III study reported a median overall survival (OS) of 22.1 months in patients with recurrent or metastatic NPC treated with gemcitabine plus cisplatin (3). The 5-year OS probability was only 31.0%. Later, checkpoint inhibitor immunotherapy was compared to chemotherapy in the KEYNOTE-122 study. The results demonstrated no improvement in OS but a reduced incidence of treatment-related adverse events with pembrolizumab monotherapy (12). Combining checkpoint inhibitor immunotherapy with chemotherapy significantly improved PFS and OS compared to chemotherapy alone. The JUPITER-02 study compared toripalimab with placebo in combination with gemcitabine-cisplatin chemotherapy for patients with recurrent or metastatic NPC who were chemotherapy-naïve. In the toripalimab group, the median PFS was 21.4 months, and median OS was not reached, compared to 8.2 months PFS and 33.7 months OS in the placebo group (5). The addition of several other checkpoint inhibitors to chemotherapy also demonstrated benefits in PFS, while OS data are still under investigation (4, 6). Consequently, the combination of immunotherapy and chemotherapy is now the first-line treatment for patients with recurrent or metastatic NPC.

However, the standard combination treatment may not be suitable for patients with NPC exhibiting extensive bone marrow metastasis. Bone marrow metastasis is a poor prognostic factor in all non-hematologic malignancies (13). Patients with bone marrow metastasis typically lack specific clinical manifestations and commonly exhibit symptoms such as anemia, thrombocytopenia, bone pain, and bleeding tendencies (14). Since bone marrow aspiration is not a routine test for solid tumors, most patients have already experienced diffuse tumor invasion in the bone marrow at the time of diagnosis. At this stage, many patients have developed fatal complications, such as severe myelosuppression, infection, and disseminated intravascular coagulation (13). This creates a treatment dilemma: these patients may struggle to tolerate the side effects of standard-dose chemotherapy. Conversely, without effective antitumor treatment, the patient’s condition will rapidly deteriorate. Given the complexities associated with bone marrow metastasis in NPC, a tailored and individualized treatment strategy is imperative.

FDG-PET/CT exhibits excellent diagnostic performance in the N and M staging of NPC (7). In patients with locally advanced squamous cell carcinoma of the head and neck, FDG-PET/CT holds significant value in altering the overall staging, determining the radiation therapy target area, and assessing the therapeutic effects (15). Conducting FDG-PET/CT promptly when there is clinical suspicion of metastasis can help improve the prognosis (16). As for bone marrow metastasis, successful and early identification of interval bone marrow oligometastasis can also be achieved through the use of FDG-PET/CT (17).

Reports on NPC with bone marrow metastasis are rare, and the prognosis for these patients is extremely poor. In 1991, Zen et al. reported five cases of NPC with bone marrow metastasis (10). The median survival of the patients was only 16 days, with the longest survivor living for only 3 months. Berry et al. reported a case with bone marrow metastasis in primary NPC, and the patient survived for only 2 weeks (9). Miyaushiro et al. reported a patient with NPC and bone marrow metastasis treated with weekly paclitaxel therapy (8). The patient showed a partial response and survived for 8 months, indicating that low-dose chemotherapy may be an option to improve patients’ conditions when they cannot tolerate standard-dose chemotherapy. To our knowledge, the best reported outcome for NPC with bone marrow metastasis comes from a case reported by Zhang et al., involving a 44-year-old male patient. He achieved symptom relief and long-term survival following an initial treatment of chemotherapy plus cetuximab and a maintenance therapy of capecitabine plus PD-1 inhibitor sintilimab. At the time of the article’s publication, the patient’s survival had reached 16 months. The case highlights the role of combination therapy (18).

Compared to chemotherapy, immunotherapy has mild hematologic toxicity and is better tolerated, facilitating the management of adverse events in patients with compromised performance status (12). In addition, preclinical studies provided evidence on the immunomodulation effect of cisplatin (19). As shown in the present case, initiating treatment with immunotherapy combined with a reduced dose of cisplatin allows for a balance between efficacy and toxicity. Once the patient’s condition improves, the chemotherapy dose can be gradually increased to the standard level, enabling further remission.

For NPC with diffuse metastasis, systemic therapy is often the first-line treatment. The present case underwent artificial femoral head replacement in another center before starting systemic antitumor treatment considering the existence of pathological fracture of right femoral neck. In our opinion, making such decisions should be prudent because the recovery from surgery can postpone prompt chemotherapy and immunotherapy. A multidisciplinary consultation should be strongly recommended in this process.

This report documents another successful case of managing bone marrow metastasis from NPC through immunotherapy-based combination therapy. As of the current report, the patient has attained an overall survival of 18 months, and continues to survive with no evidence of disease. Nevertheless, several challenges and uncertainties emerge regarding the management of such cases. Studies have shown the effectiveness of various immune checkpoint inhibitors when combined with chemotherapy in advanced NPC (4–6). However, the optimal combination remains unclear. Moreover, determining the regimen for maintenance therapy poses challenges. In most clinical trials of chemotherapy combined with immunotherapy for the treatment of advanced NPC, maintenance therapy typically involved immune checkpoint inhibitor monotherapy after its combination with 4–6 cycles of chemotherapy. However, the unmeasurable nature of bone marrow metastasis makes it difficult for early detection of disease progression. Delayed diagnosis of disease progression may render patients unable to tolerate second-line therapy due to complications from bone marrow metastasis. In our case, FDG-PET/CT scans performed 4 months before the end of chemotherapy still showed increased bone marrow FDG uptake. Given the risk of disease progression, we administered immunotherapy in combination with fruquintinib, a vascular endothelial growth factor receptor - tyrosine kinase inhibitor (VEGFR-TKI). This decision was based on preliminary evidence from a phase II study suggesting the efficacy of VEGFR-TKI plus anti-PD-1 therapy in patients with advanced NPC (20). Notably, the maintenance therapy led to complete remission in this case, with no severe adverse events occurring.

In summary, our case indicates that checkpoint inhibitor immunotherapy-based combination therapy is an effective and well-tolerated treatment for patients with NPC with bone marrow metastasis.

## Data availability statement

The raw data supporting the conclusions of this article will be made available by the authors, without undue reservation.

## Ethics statement

The studies involving humans were approved by the ethics committee of Tongren Hospital, Shanghai Jiao Tong University School of Medicine. The studies were conducted in accordance with the local legislation and institutional requirements. The participants provided their written informed consent to participate in this study. Written informed consent was obtained from the individual(s) for the publication of any potentially identifiable images or data included in this article.

## Author contributions

XW: Data curation, Investigation, Writing – original draft, Writing – review & editing. YY: Data curation, Investigation, Writing – original draft, Writing – review & editing. YZ: Investigation, Methodology, Writing – review & editing. GQ: Methodology, Resources, Writing – review & editing. SC: Resources, Writing – review & editing. WH: Resources, Writing – review & editing. JZ: Methodology, Project administration, Supervision, Writing – review & editing.
